# From the Incoming Editor

**DOI:** 10.3390/jintelligence9040051

**Published:** 2021-10-23

**Authors:** Andrew R. A. Conway

**Affiliations:** Division of Behavioral and Organizational Sciences, Claremont Graduate University, Claremont, CA 91711, USA; andrew.conway@cgu.edu

First, let me say that I am honored to take on the role of Editor in Chief at the *Journal of Intelligence*. My predecessor, Paul De Boeck, has done an excellent job at the helm for the last eight years. And it has been my privilege to work with Paul as an Associate Editor here at the journal for the last three years. My primary goal as editor is to continue that success and to build upon the strong foundation that Paul and the editorial team have provided for the *Journal of Intelligence*.

Intelligence is a complex psychological construct, and in turn, the science of intelligence is multi-faceted and interdisciplinary, including work from several broad fields, such as developmental psychology, cognitive psychology, psychometrics, behavioral genetics, and neuroscience. The *Journal of Intelligence* has now published 252 articles, and taken together, these 252 papers reflect the multi-faceted nature of the field. The journal has published papers on an extremely broad range of topics, from mitochondrial functioning to group problem solving.

In my view, the journal has already established a strong reputation in three areas: The application of cognitive models to the study of intelligence, psychometric network modeling, and the exploration of relatively novel concepts and constructs in the study of individual differences. I am enthusiastic about all of these areas of research, and I expect the journal to continue to build upon these strengths and be an outlet for more work along these lines.

At the same time, I believe there are some opportunities for broadening our scope. In particular, I would like to see the journal publish more developmental research, both theoretical and applied. I therefore encourage developmental, educational, and school psychologists to consider the *Journal of Intelligence* as an outlet for their work. I would also like to see the journal publish more research at the intersection of cognitive neuroscience and psychometrics and therefore encourage research teams that bridge these two disciplines, as well as cognitive neuroscientists who do individual differences studies, to consider the *Journal of Intelligence* as an outlet for their work.

As I said in 2014 (https://doi.org/10.3390/jintelligence2020033), when the journal was just beginning, advances in psychometrics, cognitive psychology, and neuroscience set the stage for the development of stronger theories and more sophisticated models of intelligence. Having an open access journal as an outlet for this new research has been crucial. I am excited to see what new findings and ideas emerge in the coming years.

